# Fullerene Derivatives for Drug Delivery against COVID-19: A Molecular Dynamics Investigation of Dendro[60]fullerene as Nanocarrier of Molnupiravir

**DOI:** 10.3390/nano12152711

**Published:** 2022-08-07

**Authors:** Georgios I. Giannopoulos

**Affiliations:** Department of Mechanical Engineering, University of Peloponnese, 1 Megalou Alexandrou Street, GR-26334 Patras, Greece; ggiannopoulos@uop.gr

**Keywords:** fullerene, drug delivery, COVID-19, molnupiravir, molecular dynamics, solvation free energy

## Abstract

In this paper, a theoretical investigation is made regarding the possibility of using a water-soluble derivative of C_60_ as a drug delivery agent for treating Coronavirus disease 2019 (COVID-19). Molnupiravir is chosen as the transporting pharmaceutical compound since it has already proved to be very helpful in saving lives in case of hospitalization. According to the proposed formulation, a carboxyfullerene known as dendro[60]fullerene is externally connected with two molnupiravir molecules. Two properly formed nitrogen single bonds (N−N) are used as linkers between the dendro[60]fullerene and the two molnupiravir molecules to create the final form of the C_60_ derivate/molnupiravir conjugate. The energetics of the developed molecular system and its interaction with water and n-octanol are extensively studied via classical molecular dynamics (MD) using the COMPASS II force field. To study the interactions with water and n-octanol, an appropriate periodic amorphous unit cell is created that contains a single C_60_ derivative/molnupiravir system surrounded by numerous solvent molecules and simulated via MD in room conditions. In addition, the corresponding solvation-free energies of the investigated drug delivery system are computed and set in contrast with the corresponding properties of the water-soluble dendro[60]fullerene, to test its solubility capabilities.

## 1. Introduction

The COVID-19 pandemic has driven the majority of recent research efforts in the rapid development of new vaccines [1], antibody drugs [2], and antiviral medicines [3] against the SARS-CoV-2 virus. Nanotechnology and its discoveries in the last few decades could not be absent in this challenge [4].

In the field of drugs, perhaps one of the most promising medicines is the so-called molnupiravir, which seems that it may contribute to the healing from COVID-19 and its symptoms when given orally in solid-dose forms such as tablets and capsules [5], which provide a good degree of stability and deliver precise dosage. The molnupiravir reduces the ability of SARS-CoV-2, the virus that causes COVID-19, to multiply in the body [6]. Very recently, Sharov et al. [7] provided valuable density functional theory (DFT) calculations that may contribute to the understanding and development of new therapies against SARS-CoV-2 based on molnupiravir. In their in-depth computational study, they performed molecular docking simulations of three plausible tautomeric forms of molnupiravir with a series of the SARS-CoV-2 proteins to predict the corresponding structural, electronic, and optical responses. Given the action mechanism of molnupiravir, it becomes evident that the more controllable and accurate the delivery of the specific drug within the human body, the more successful the provided treatment against COVID-19. In this effort, the drug delivery approaches and technologies via the use of nanoparticles [8] may help to achieve the desired therapeutic results against COVID-19 since they may provide safe storage and transport of the required pharmaceutical compound to its target location with a high degree of precision.

It is now well understood that the most critically affected human organ by SARS-CoV-2 is the lung [9]. Therefore, there is already an open discussion about the optional strategies to prevent or fight the action of the virus in the deep lung and to target the most important host cells via more focused drug delivery strategies [10]. In any case, there are certain areas within the human body where the virus-cell populations are denser or spots where the potential damage and risk are much higher [11] and, thus, it seems that drug delivery methods that are based on nanostructured systems [12] may offer very promising treatment solutions against the recent Coronavirus disease. In an extensive review, Abd Elkodous et al. [13] gave insights into several nanomaterial-based methodologies for increasing the effectiveness of the drugs against COVID-19 by their attachment inside or onto the molecular surface of five promising drug delivery nanostructured agents, i.e., nano-emulsions, liposomes, polymeric nanoparticles and micelles, dendrimers, and nano-suspensions. Similarly, Witika et al. [14] provided a state-of-the-art regarding the possible use of biomimetic nanocarriers for the efficient treatment of COVID-19. They have indicated that there is a great potential for adopting a variety of nanomaterials such as nanospheres, nanocrystals, solid lipid nanoparticles, nanosponges, etc. to enable effective targeted delivery of chemical substances to specific cells. In a more focused attempt, Thakur et al. [15] discussed the concept of respiratory delivery of favipiravir-tocilizumab combination through mucoadhesive protein-lipidic nanovesicles as a therapeutic tactic against COVID-19.

The carbon-based nanomaterials due to their exceptional structural and physical properties have attracted much of the research interest for use in biomedicine and, especially, in drug delivery technologies. Above the various allotropes of carbon, fullerenes [16] have been proposed as promising candidates for drug carriers, mainly because of their unique spherical molecular shape. There are numerous experimental [17,18,19,20,21,22,23] as well as theoretical reports [24,25,26,27,28,29,30,31,32,33], mainly grounded on DFT, associated with the analysis of drug delivery systems made of fullerene derivatives. Nevertheless, the relative computational approximations and predictions regarding the feasibility of using carbon nanomaterials as vehicles for anti-SARS-CoV-2 drugs are much fewer due to the freshness of the corresponding pandemic. In the field, Shahabi and Raissi [34], using Molecular Dynamics (MD) as a computational tool, conducted simulations of the transfer and delivery of the anti-SARS-CoV-2 drug Carmofur with the assistance of graphene oxide quantum dots. It was found that graphene oxide quantum dots offer efficient drug delivery around the catalytic region of the COVID-19 protein while promoting good drug penetration at the target location. Based on DFT instead, Bibi et al. [35] investigated the fullerene C_60_ doped with metals Cr, Fe, and Ni as the carrier of the favipiravir anti-COVID-19 drug. In an analogous attempt, Yao et al. [36] explored the potential of using fullerene-like metal oxide nanocages as drug-carrying systems for favipiravir by estimating their drug adsorption efficiency. On the other hand, Parlak et al. [37] investigated the possibility of using a silicon-doped C_60_ as a drug delivery carrier of molnupiravir by using DFT simulations. Specifically, they developed and optimized a SiC_59_ molecular structure and, then, assumed that its silicon atom is externally bonded with an atom of a single molnupiravir molecule. They examined the bonding of the silicon atom of the doped fullerene with six different atoms of the drug molecule in order to find the ideal intermolecular interaction and optimum linking.

In the present study, a molecular system based on an appropriate cyclopropane derivative of C_60_ fullerene was proposed and tested as a carrier agent of the molnupiravir drug. The idea is to use a functionalized molecular structure of C_60_ fullerene, which is well soluble in water. In this way, not only the excellent and already proven drug-carrying capabilities of C_60_ will be taken advantage of, but also the obtained system is expected to present good solubility, which is a key property and a required characteristic for drug delivery nanosystems. The MD simulations and corresponding energetic calculations are carried out to characterize the structural behavior and stability of the investigated water-soluble C_60_ derivative/molnupiravir system. A carboxyfullerene known as dendro[60]fullerene is chosen as the basic structure of the delivery system, a molecule that was synthesized several years ago [38] and has already proved its good solubility in water [38,39]. In order to develop the proposed system, two molnupiravir molecules are attached to the two major building blocks of the dendro[60]fullerene via N-N single bonding. To test if the new drug delivery system has the ability to form a solution with water, the Gibbs free energy of solvation [40] is calculated using suitable MD modeling and simulations. In order to reach clear conclusions, the dendro[60]fullerene alone is also tested in water and n-octanol environments to be compared with the proposed dendro[60]fullerene/molnupiravir molecules drug delivery system. Despite the fact that there are several reports [28,35,37,41,42,43,44,45] on the exploitation of the use of C_60_-based molecular systems against COVID-19, to the author’s best knowledge, this is the first time that dendro[60]fullerene has been proposed and tested as a possible nanovehicle of molnupiravir.

## 2. Solubility and Partition Coefficient

A crucial design factor for many biochemical procedures and operations is the relative solubility of a substance in an aqueous and an organic solvent, which is usually expressed by the partition coefficient *P*. The partition coefficient *P* describes the tendency of an uncharged molecule to better dissolve in water or lipids and consequently to have a hydrophilic/lipophobic or lipophilic/hydrophobic nature, respectively. More simply, it defines the amount of a solute that dissolves in the water portion versus an organic portion. The most commonly followed tactic is to make estimations of the partitioning between water and n-octanol. In addition, it is more convenient to express the resulting partitioning by providing the constant log*P*, which is equal to the decimal logarithm of the partition coefficient *P* as follows:

(1) $$\log P=\log\frac{\left[\mathrm{octanol}\right]}{\left[\mathrm{water}\right]}$$

where [octanol] and [water] denote the concentration of solute in the n-octanol and water, respectively.

It should be noted that obtaining a negative log*P* constant implies that the investigated compound is more hydrophilic, more polar, and has poorer lipid bilayer permeability. In contrast, a positive value for log*P* leads to the conclusion that the compound is more lipophilic, more nonpolar, and has poor aqueous solubility. Finally, when log*P* is close to zero, the molecule under investigation is similarly partitioned between the lipid and aqueous phases. The above short analysis makes clear that log*P* may be used extensively as a prognosticator of the interactions of drug components and products in the human body, which is separated into aqueous as well as lipid regions. Therefore, the log*P* constant may define the delivery and distribution of the drug molecules between the aqueous phase outside the human cell’s membrane and the lipid phase inside the cell’s membrane.

## 3. Solubility and Solvation Free Energy

The solubility of a compound may be expressed with respect to free energy by the difference of the Gibbs free energies [40] of the neutral form of a compound species in two different solvents, α, and β, as follows:

(2) $$\Delta G=\Delta G^{\mathrm{solv},\alpha }-\Delta G^{\mathrm{solv},\beta }=RT\ln\left(P_{\alpha \to \beta }\right)$$

or

(3) $$\Delta G=RT\ln\left(10\right)\log\left(P_{\alpha \to \beta }\right)$$

where *R* = 1.987 × 10^−3^ kcal/mol/K is the gas constant, *T* is the temperature in K, and *P*_α→__β_ is the partition coefficient defined as the ratio of the number concentrations of the solute in the phases α and β, respectively.

A very common method for approximating the free energy between subsequent states is to computationally calculate the derivative of the total energy, including both the potential and kinetic energy of the studied system. This theoretical approach is known as “thermodynamic integration” and is commonly used for free energy calculations based on computer simulations [46]. According to this numerical technique, the influence of the interaction between the solute and the solvent is progressively increased from zero to full scale in a finite number of steps. A complete molecular simulation must be performed separately for each step in order to compute the corresponding derivative of the solvation-free energy. When all simulations are finalized, the free energy may be defined by the numerical integration of all calculated derivatives. A very brief mathematical representation of the method is given below.

Let us assume a pair of end states α and β and that their corresponding Hamiltonians, i.e., total energies including both potential and kinetic energies, are denoted as *H*_α_(**q**, **p**; *λ*) and *H*_β_(**q**, **p**; *λ*), where **q** and **p** represent all of the positions and momenta of the system while *λ* is a coefficient taking values from 0 to 1. A total Hamiltonian *H* may be defined as follows:

(4) $$H=f(\lambda )H_{\alpha }(q,p;\lambda )+g(\lambda )H_{\alpha }(q,p;\lambda )$$

where *f* and *g* are functions of *λ* that are utilized to mix the two Hamiltonians, which are generally chosen such that *H* = *H*_α_ for *λ* = 0 and *H* = *H*_β_ for *λ* = 1.

The Gibbs free energy difference between α and β states may now be calculated by the following relationship:

(5) $$\Delta G=\int_{\lambda =0}^{\lambda =1}{\langle \frac{\partial H}{\partial \lambda }\rangle }_{\lambda }d\lambda$$

The above integral may be approximated by performing a series of simulations regarding a discrete set of *λ* values and then adopting a numerical quadrature approach.

The calculation of the solvation-free energy is performed in three simulation stages. First, the ideal contribution is computed. During this stage, the solute molecule is assumed to be in a vacuum while the charges are steadily reduced to zero, whereas the rest of the interactions are kept the same. Second, the van der Waals contribution is calculated. In this kind of simulation process, the chargeless molecule is coupled to the solvent by activating the van der Waals interaction in several steps. The final stage is related to the approximation of the electrostatic contribution. Here, the charges of the solute molecular system are regenerated while the solute compound is progressively immersed in the solvent. Evidently, the total solvation free energy is equal to the sum of the ideal, van der Waals, and electrostatic aforementioned contributions.

## 4. Simulation Details

The present theoretical investigations are performed by utilizing force-field-based MD. The specific numerical technique has been proved to be reliable, and efficient, followed by a reasonable computational cost when used for studying pharmaceutical and drug applications at the molecular level [47]. The proposed MD simulations are grounded on the COMPASS II (Condensed-phase Optimized Molecular Potentials for Atomistic Simulation Studies II) [48,49] based force-field, which consists of terms for bond stretching (*b*), angle bending (*θ*), dihedral angle rotating (*φ*), out-of-plane angle distortion (*χ*) as well as cross-terms that account for interactions between the above four types of internal coordinates, and two non-bonded functions, a Coulombic function for electrostatic interactions due to the charges *q* and a 9-6 Lennard-Jones potential for van der Waals (vdW) interactions. The general form of the utilized potential is given by the following:

(6) $$E_{\mathrm{total}}=E_{b}+E_{\theta }+E_{\phi }+E_{\theta }+E_{\chi }+E_{b,b’}+E_{b,\theta }+E_{b,\phi }+E_{\theta ,\theta ’}+E_{\theta ,\theta \prime ,\phi }+E_{q}+E_{\mathrm{vdW}}$$

or in a more analytical form as follows:

(7) $$\begin{array}{l} E_{\mathrm{total}}=\sum_{b}[k_{2}{\left(b-b_{0}\right)}^{2}+k_{3}{\left(b-b_{0}\right)}^{3}+k_{4}{\left(b-b_{0}\right)}^{4}]+\\ +\sum_{\theta }[k_{2}{\left(\theta -\theta_{0}\right)}^{2}+k_{3}{\left(\theta -\theta_{0}\right)}^{3}+k_{4}{\left(\theta -\theta_{0}\right)}^{4}]+\\ +\sum_{\phi }\left[k_{1}(1-\cos\phi \right)+k_{2}(1-\cos2\phi )+k_{3}(1-\cos3\phi )]+\sum_{\chi }k_{2}\chi^{2}+\\ +\sum_{b,b’}k(b-b_{0})(b^{\prime }-{b^{\prime }}_{0})+\sum_{b,\theta }\left[k(b-b_{0})(\theta -\theta_{0}\right)+\\ +\sum_{b,\varphi }(b-b_{0})[k_{1}\cos\phi +k_{2}\cos2\phi +k_{3}\cos3\phi ]+\\ +\sum_{\theta ,\varphi }(\theta -\theta_{0})[k_{1}\cos\phi +k_{2}\cos2\phi +k_{3}\cos3\phi ]+\sum_{\theta ,\theta^{\prime }}k(\theta -\theta_{0})(\theta^{\prime }-{\theta^{\prime }}_{0})+\\ +\sum_{\theta ,\theta^{\prime },\varphi }k(\theta -\theta_{0})(\theta^{\prime }-{\theta^{\prime }}_{0})\cos\phi +\sum_{i,j}\frac{q_{i}q_{j}}{r_{ij}}+\sum_{i,j}\varepsilon_{ij}\left[2{\left(\frac{r_{ij0}}{r_{ij}}\right)}^{9}-3{\left(\frac{r_{ij0}}{r_{ij}}\right)}^{6}\right] \end{array}$$

In the above equation, *r_ij_* = *r_i_* − *r_j_* is the distance between atoms *I* and *j* having the atomic positions *r_i_* and *r_j_*, respectively. The subscripts 0 in the above parameters represent the corresponding reference values. The stiffness-like coefficients *k*, *k*_1_, *k*_2_, *k*_3_ and *k*_4_ are the COMPASS-II force field constants. The parameters *q_i_* and *q_j_* are the charges of atoms *i* and *j*, respectively. Finally, the functional terms *r_ij_*_0_ and *ε_ij_* are given, respectively, by the following:

(8) $$r_{ij0}={\left(\frac{r_{i0}^{6}-r_{j0}^{6}}{2}\right)}^{1/6}$$

(9) $$\varepsilon_{ij}=2\sqrt{\varepsilon_{i}\cdot \varepsilon_{j}}\left(\frac{r_{i0}^{3}\cdot r_{j0}^{3}}{r_{i0}^{6}+r_{j0}^{6}}\right)$$

where *ε_i_* and *ε_j_* are the van der Waals well depths of the atoms *i* and *j*, correspondingly.

For the purpose of the present investigation, the Andersen thermostat and Berendsen barostat are adopted to control, respectively, the temperature and pressure of the system during the simulations [50]. In addition, the group-based summation method with cut-off radii of 12.5 Å and long-range corrections are assumed for the estimations of the vdW and the electrostatic interactions [50]. In all numerical tests, it is considered that the environment of the unit cells is characterized by a room temperature of *T* = 298 K and, if required, a pressure of *p* = 1 atm. The optimization of the unit cells is achieved by using the steepest descent algorithm under a convergence tolerance of 2 × 10^−5^ kcal/mol, 10^−3^ kcal/mol/Å, and 10^−5^ Å regarding the energy, force, and distance variation, respectively.

## 5. Proposed Drug Delivery System

Because of their great physical properties, fullerenes have proved to be ideal nanomaterials in many biological applications [51]. Especially the Buckminsterfullerene C_60_, due to its symmetric hollow spherical shape, shares unique attributes and, thus, has gained much attention in the field of biosciences. The compact size of the fullerene C_60_ makes it an ideal nanovehicle for drug delivery applications. Moreover, this nanomaterial, because of its physicochemical properties and size, is optimally suited to enter deep into the lung through the airways.

**Figure 1:**
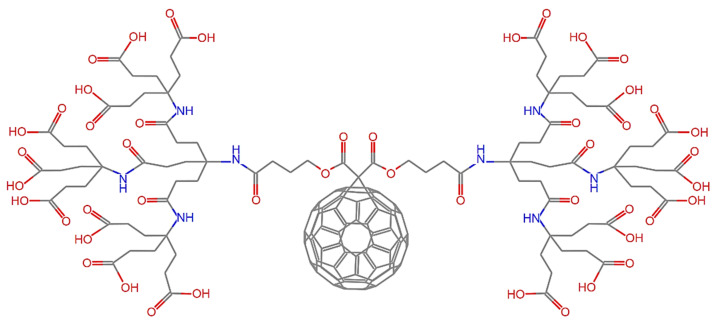
The chemical structure of the water-soluble dendro[60]fullerene.

Regarding the possibility of using C_60_ for carrying drug molecules in the human body, one important disadvantage is the fact that it presents significant hydrophobic behavior. In view of this limitation, here, a water-soluble C_60_ derivative is proposed as a nano-carrier. Specifically, in order to enhance solubility and avoid aggregation during delivery, the dendro[60]fullerene is adopted as the basic structure of the drug delivery system, which is shown in Figure 1. The specific dendritic monoadduct of C_60_ has 18 carboxylic groups in the periphery and may be synthesized by nucleophilic cyclopropanation of C_60_ with a bis(polyamide)-malonate dendrimer and subsequent deprotection of the terminal t-butyl groups [38]. Generally, the higher the number of carboxylic acid groups, the higher the water solubility.

**Figure 2:**
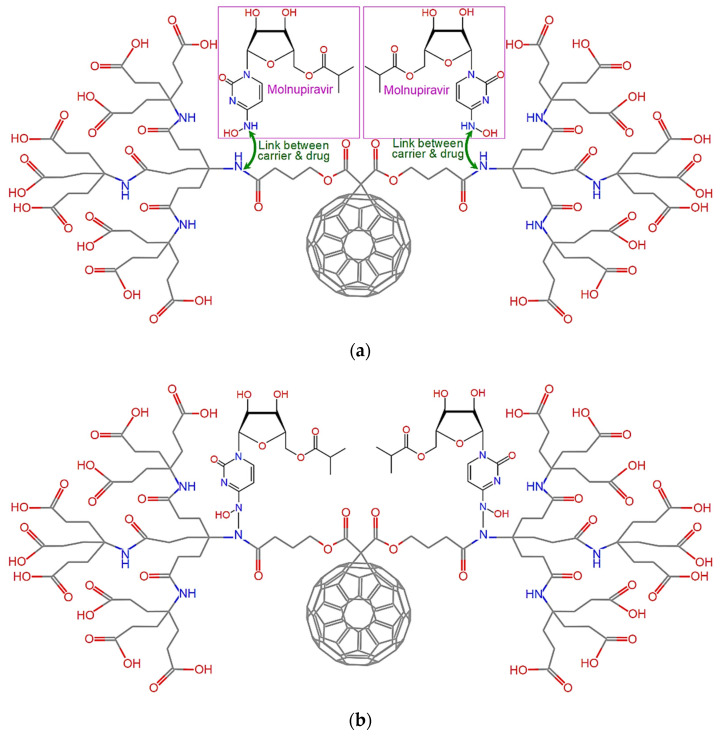
The proposed drug delivery system: (**a**) Locations of molnupiravir molecules input in dendro[60]fullerene and (**b**) final chemical structure.

The next step is the selection of an efficient drug against COVID-19, capable of being carried and delivered by dendro[60]fullerene. The drug molecule that has been preferred is molnupiravir, an antiviral drug officially indicated for the treatment of COVID-19 [5,6]. Special attention is required regarding the link between the proposed nanovehicle and the drug molecule/es. The use of smart linkers between the two compounds that may respond and react due to a stimulus is very crucial for the effective on-site and on-purpose release of the drug [52]. The ideal linker selection requires substantial experimental effort using a variety of environmental conditions, representing the different human body regions. In this study, the main idea is to adopt input positions for the drug that offer simple linkage as well as unique and sensitive response properties in the connection region. The most convenient way to achieve these two features is to use a simple bond as a linker, which is characterized by a distinctively small dissociation energy. The introduction of a linkage of small strength in comparison with the rest of the bonds in the system will allow the easy and controllable release of the drug molecules, avoiding structural modifications of any of the two coupled basic compounds at the drug release instant. By observing in Figure 2a the dendro[60]fullerene in contrast with the molecular structure of the molnupiravir, one may notice that both compounds contain similar −N−H (−Nitrogen−Hydrogen) subunits. Thus, it becomes evident that the single N−N bond could be proposed and tested as a possible simple linker at these locations, as depicted in Figure 2b. To retain the molecular symmetry of the carrier, two corresponding −N−N− links are created with two drug molecules in opposing positions. Although the dendro[60]fullerene contains 6 more −N−H subunits in its periphery, drug molecule inputs in these sites are avoided, in order not to disturb the dendritic formations at the two edges of the system, which would probably harm its overall solubility.

Although the N–N bond has a relatively small dissociation energy of 240 kJ/mol, this value is only 20% smaller than that corresponding to the C–N bond, which is the next weakest link existing in the proposed system [53]. In addition, it is worth mentioning at this point that over the last few years, plenty of reactions capable of forming the nitrogen−nitrogen bond in natural product biosynthesis have been revealed, overcoming the technical difficulties due to the inherent nucleophilic nature of nitrogen elements [54]. Moreover, some nitrogen–nitrogen-bond-containing bioactive products have already been used in clinical practice, evidencing their promising long-life capabilities [54].

## 6. Results and Discussion

The main scope of the computational study is the characterization of the solubility of the proposed dendro[60]fullerene/molnupiravir drug delivery system through solvation-free energy MD computations. For comparison, the pure water-soluble dendro[60]fullerene is computationally investigated in parallel. Both compounds are tested in water and n-octanol (1-octanol) to enable calculations via Equation (3).

**Figure 3:**
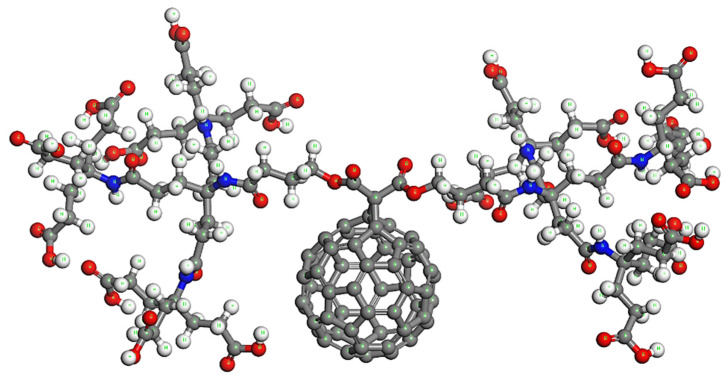
The optimized molecular structure of the dendro[60]fullerene.

**Figure 4:**
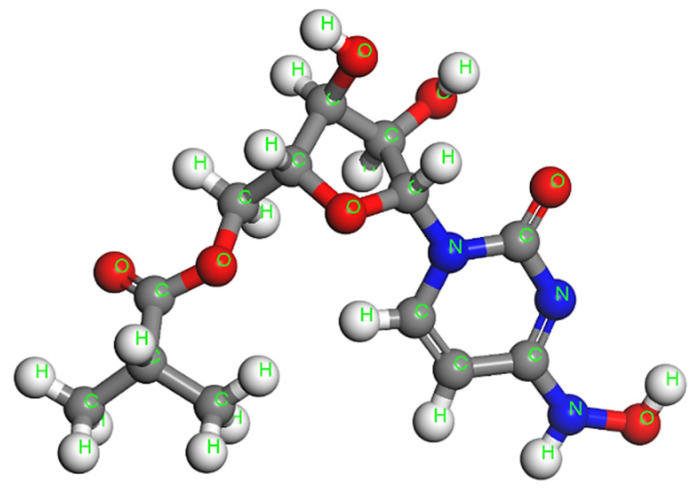
The optimized molecular structure of the molnupiravir.

**Figure 5:**
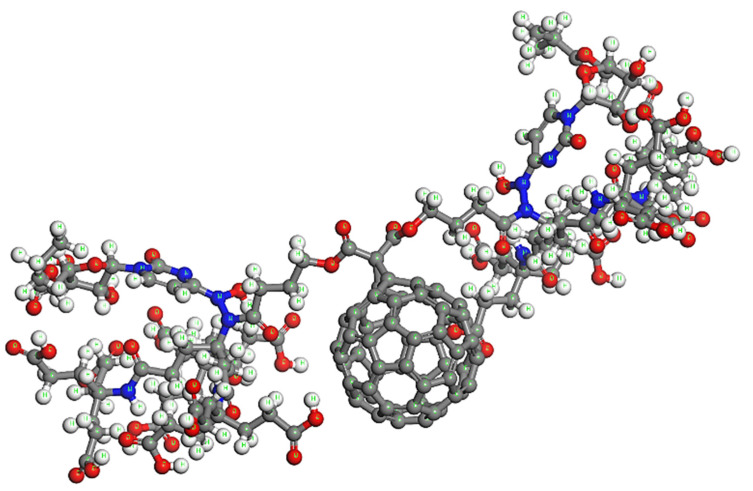
The optimized molecular structure of the dendro[60]fullerene with two molnupiravir molecules attached in the opposing dendrimer building blocks.

Firstly, all the molecular units are optimized at room temperature using the steepest descent algorithm and COMPASS II potential to obtain the corresponding lowest energy stable structures. Figure 3 illustrates the optimized molecular structure of the pure dendro[60]fullerene. The obtained optimized potential energy of this C_60_ derivative is *E*_C60der_ = −1882.09 kcal/mol. In addition, Figure 4 shows the equilibrated three-dimensional atomistic structure of a single molnupiravir molecule, which leads to a potential energy value of *E*_Molnup_ = −39.28 kcal/mol. Two optimized molnupiravir molecules are appropriately attached to the two major building blocks (see Figure 2) of the adopted optimized nanovehicle. A new optimization process is carried out for the entire system this time, which leads to the molecular structure of Figure 5. The arisen optimized drug delivery molecular system that is created via the connection of the dendro[60]fullerene and two molnupiravir molecules via nitrogen-nitrogen bonding has a total energy of *E*_drugsys_ = −2008.80 kcal/mol. According to the above energy values, the binding energy between the nanocarrier and the two drug molecules is equal to *E*_bind_ = *E*_drugsys_ − (*E*_C60der_ + 2*E*_Molnup_) = −48.15 kcal/mol, the negative value of which indicates that the interaction between the nanocarrier and the drug is reasonably stable.

**Figure 6:**
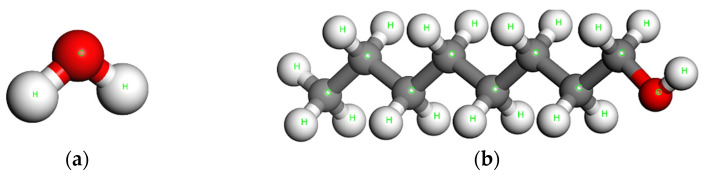
The utilized solvent particles after being optimized: (**a**) Water and (**b**) n-octanol (1-octanol).

Next, representative unit cell models are constructed for the evaluation of the interactions of the dendro[60]fullerene (Figure 3) and the dendro[60]fullerene/molnupiravir system (Figure 5) in water and n-octanol solvents. As expected, for the solute molecules presented in Figure 3 and Figure 5, the optimized state of the solvent molecules should be obtained as well. The steepest descent optimization leads to the equilibrium molecular structures of water and n-octanol, which are illustrated in Figure 6a,b, respectively.

**Figure 7:**
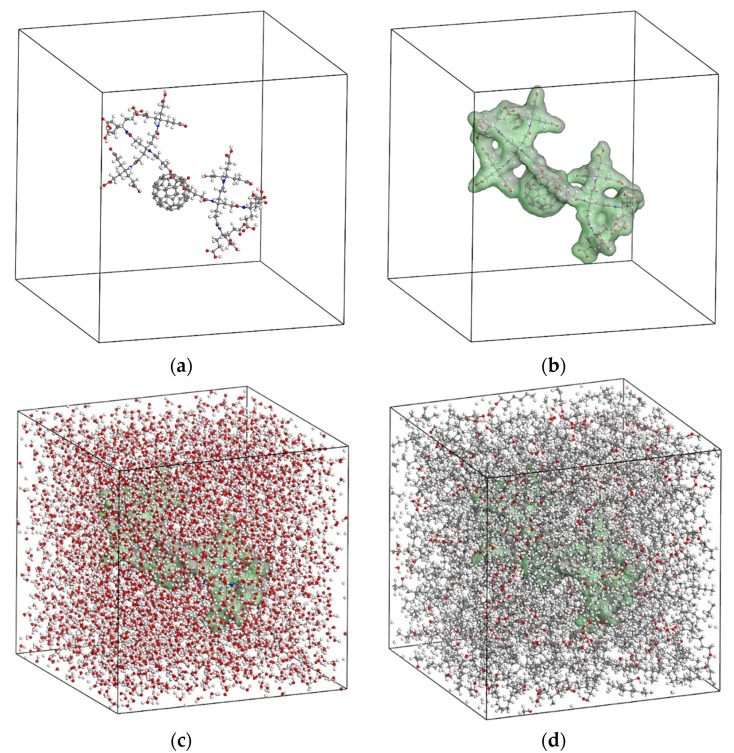
The unit cell for simulating the dendro[60]fullerene as a solute: (**a**) Solute central position, (**b**) solute Connolly surface, (**c**) solute in water solvent, and (**d**) solute in n-octanol solvent.

**Figure 8:**
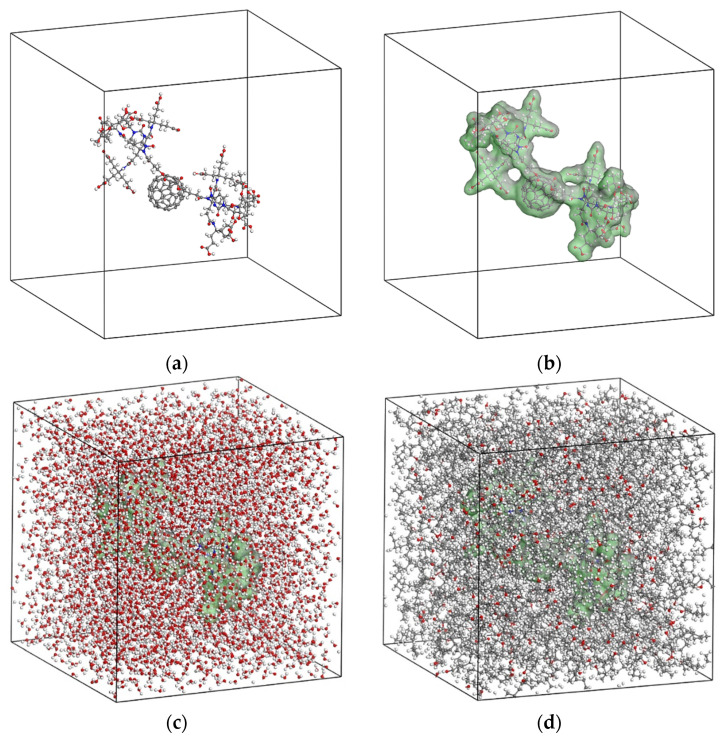
The unit cell for simulating the dendro[60]fullerene/molnupiravir drug delivery system as a solute: (**a**) Solute central position, (**b**) solute Connolly surface, (**c**) solute in water solvent, and (**d**) solute in n-octanol solvent.

The representative amorphous simulation box for each of the investigated solvent/solute systems is generally constructed as described below. Initially, a periodic and symmetric cubic unit cell with a centrally positioned single solvent molecule is developed by assuming a large enough lattice length of 50 nm. The cubic unit cells containing the dendro[60]fullerene and the drug delivery molecular system are illustrated in Figure 7a and Figure 8a, respectively. Then, a Connolly surface is created to define the boundary between the solvent molecular structure and its environment, which is going to be filled with solute molecules. The developed Connolly surfaces for the dendro[60]fullerene and the dendro[60]fullerene/molnupiravir compound may be seen in Figure 7b and Figure 8b.

**Figure 9:**
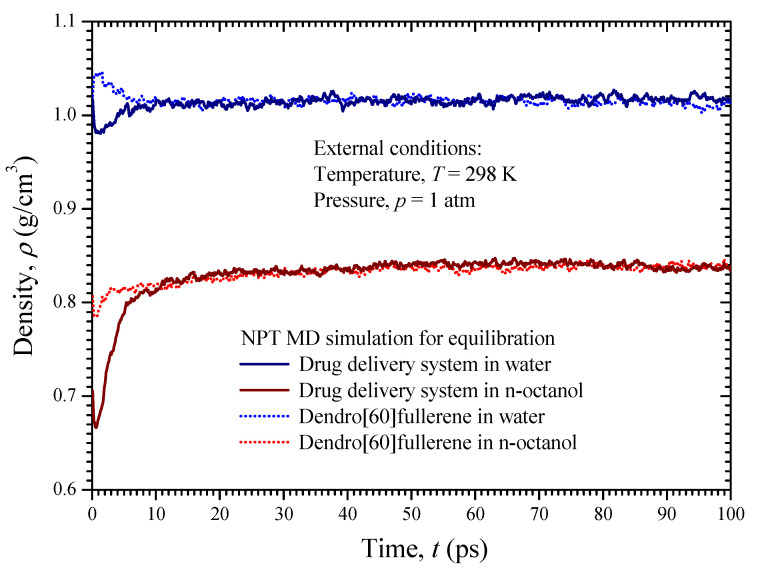
The density of the four investigated unit cells versus time during the NPT MD analysis.

The remaining empty volume in the simulation box is packed with a number of solvent molecules. The population of the solvent molecules is progressively increased in the empty unit cell volume until the theoretical density of the solvent under consideration is reached. Note that a density value of 1 g/cm^3^ and 0.824 g/cm^3^ is assumed for the water and n-octanol phase, respectively. In addition, a geometry optimization procedure is carried out followed by energy minimization until a stable structure of the representative simulation box is achieved. To further relax the simulation boxes and to obtain the true density of the solute/solvents systems, a dynamics analysis is performed using the NPT ensemble and a time step of 1 fs. An external pressure of 1 atm and a temperature of 298 K are applied for 100 ps to equilibrate the simulation boxes under normal environmental conditions. Figure 9 illustrates the density variation of the unit cells over 100 ps for the four tested solute/solvent combinations. The results show that both dendro[60]fullerene and the drug delivery molecular system when immersed in water and n-octanol converge to a density value of about 1.15 g/cm^3^ and 0.84 g/cm^3^, respectively. The equilibrated and relaxed unit cells of the dendro[60]fullerene and the dendro[60]fullerene/molnupiravir compound in water after the 100 ps NPT MD simulation are illustrated in Figure 7c and Figure 8c, respectively, whereas Figure 7d and Figure 8d present corresponding frames regarding the n-octanol case.

To compute the Gibbs free energies at room temperature through Equation (5), a series of simulations for the four unit cells of Figure 7c,d and Figure 8c,d is performed by increasing the coupling coefficient *λ* from 0 to 1 using a stable increment of Δ*λ* = 0.05. Note that for each new increment of the coupling coefficient, a full independent MD analysis is performed for 100 ps under the NVT ensemble, to achieve the required equilibration of the system under consideration. A time step of 1 fs is adopted for all simulations. At the end of each NVT analysis, an optimization process is carried out to further minimize, if possible, the potential energy of the system. The simulations for each unit cell are associated with the separate computation of the ideal energy, van der Waals, and electrostatic contribution. As already mentioned, the sum of these contributions expresses the corresponding Gibbs free energy. Given the above simulation details, it becomes evident that the free energy calculations via the MD method require a substantial computational effort.

**Figure 10:**
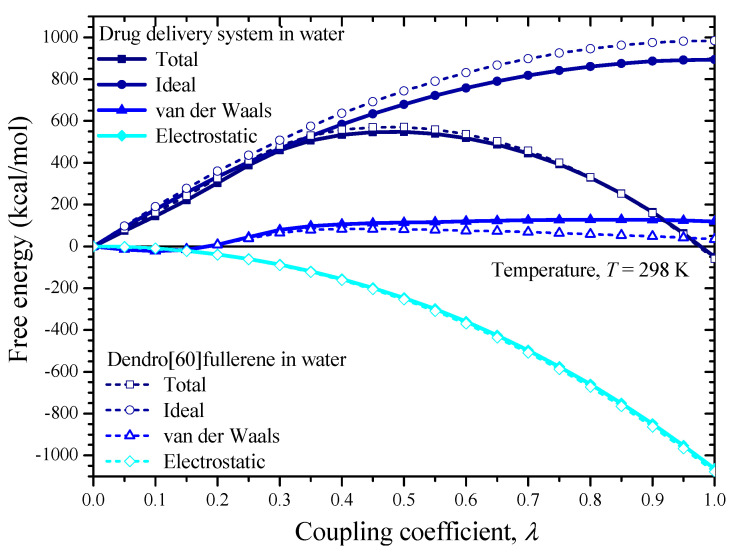
The free energies of the dendro[60]fullerene/molnupiravir drug delivery system and the dendro[60]fullerene in water versus the value of the coupling coefficient.

**Figure 11:**
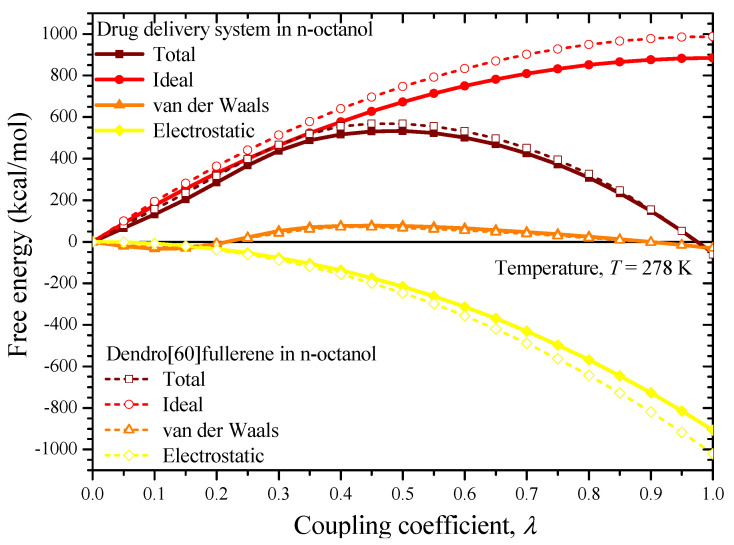
The free energies of the dendro[60]fullerene/molnupiravir drug delivery system and the dendro[60]fullerene in n-octanol versus the value of the coupling coefficient.

Figure 10 and Figure 11 illustrate the free energy contributions (total, ideal, van der Waals, electrostatic) of the two considered solutes, i.e., the drug delivery system as well as the water-soluble dendrimer fullerene, with respect to the coupling parameter increase in the water and the n-octanol, respectively. Evidently, the final free energy values correspond to *λ* = 1.

As implied by the data of both diagrams in Figure 10 and Figure 11, for all the investigated solute/solvent system cases, the ideal contribution of the solvation-free energy is very close to the absolute value of the electrostatic contribution. This occurs because the ideal contribution is actually the free energy cost of removing charges from the solute molecular system. On the other hand, van der Waals’s contribution gets lower values while it presents a mixed ascending/descending behavior as the coupling coefficient is increased. The Gibbs free energy of the drug delivery system is about −51.15 kcal/mol and −53.14 kcal/mol in water and n-octanol, respectively, meaning that it is somewhat more soluble in n-octanol than in water. On the other hand, the dendro[60]fullerene presents solvation-free energy of −59.52 kcal/mol and −62.33 kcal/mol in water and n-octanol respectively. The hydration free energy of dendro[60]fullerene (−59.52 kcal/mol) is more negative than the −51.15 kcal/mol value, a fact that proves that it has a slightly higher water-solubility compared to the same compound with two additional molnupiravir molecules on its structure. By substituting the above computed Gibbs free energy values into Equation (3), one may find that the constant logP for the proposed drug delivery system and the dendro[60]fullerene is equal to 1.46 and 2.06, respectively. These values prove that the proposed drug-delivery system not only retains the good water-solubility of the nanocarrier alone but seems to be even less lipophilic, suggesting that it is a good candidate for delivering the antiviral product molnupiravir in certain regions of the human body. Note that the unmodified C_60_ fullerene molecule, which is highly lipophilic, according to experimental evidence presents an octanol-water partition coefficient of about 6.67 [55]. Unfortunately, since dendro[60]fullerene joined with molnupiravir is studied here for the first time, more focused comparisons with other relevant available studies [28,35,37,41,42,43,44,45] are not possible for the time being.

## 7. Conclusions

A more lipophilic drug delivery system against COVID-19 may be sequestered by fatty tissue and therefore difficult to excrete and reach its target for drug release. For this reason, a new idea for a drug delivery system has been proposed here. According to the proposed concept, a highly water-soluble derivative of the C_60_, i.e., the dendro[60]fullerene has been used as a nanocarrier and two molnupiravir molecules as the drug agents. The hydrophilic nanocarrier has been linked with two drug molecules via simple N−N bonds, which may easily break under a stimulus due to their low dissociation energy. This would permit the effective synchronized release of the drug to the required target organs. The MD-based computations of the solvation free energy of the proposed drug delivery system, in contrast to the dendro[60]fullerene alone, proved the effective solubility of the proposed nanovehicle. More theoretical and experimental research is required in order to validate and adjust the proposed C_60_ derivative-based drug delivery scheme.

## Data Availability

Not applicable.
